# Senescence detection using reflected light

**DOI:** 10.1111/acel.14295

**Published:** 2024-08-05

**Authors:** Benjamin Dedic, Leo Westerberg, Andrea Mosqueda Solís, Kyle D. Dumont, Jorge L. Ruas, Anders Thorell, Erik Näslund, Kirsty L. Spalding

**Affiliations:** ^1^ Department of Cell and Molecular Biology Karolinska Institutet Stockholm Sweden; ^2^ Department of Biosciences and Nutrition Karolinska Institutet Stockholm Sweden; ^3^ Molecular and Cellular Exercise Physiology, Department of Physiology and Pharmacology Karolinska Institutet Stockholm Sweden; ^4^ Department of Pharmacology and Stanley and Judith Frankel Institute for Heart and Brain Health University of Michigan Medical School Ann Arbor Michigan USA; ^5^ Department of Clinical Sciences Danderyd Hospital, Karolinska Institutet and Department of Surgery, Ersta Hospital, Karolinska Institutet Stockholm Sweden; ^6^ Department of Clinical Sciences Danderyd Hospital, Karolinska Institutet Stockholm Sweden

**Keywords:** adipocytes, beta‐galactosidase, brightfield, reflected light, SABG, senescence, western blot, X‐gal

## Abstract

Senescence is an important cellular program occurring in development, tissue repair, cancer, and aging. Increased senescence is also associated with disease states, including obesity and Type 2 diabetes (T2D). Characterizing and quantifying senescent cells at a single cell level has been challenging and particularly difficult in large primary cells, such as human adipocytes. In this study, we present a novel approach that utilizes reflected light for accurate senescence‐associated beta‐galactosidase (SABG) staining measurements, which can be integrated with immunofluorescence and is compatible with primary mature adipocytes from both human and mouse, as well as with differentiated 3T3‐L1 cells. This technique provides a more comprehensive classification of a cell's senescent state by incorporating multiple criteria, including robust sample‐specific pH controls. By leveraging the precision of confocal microscopy to detect X‐gal crystals using reflected light, we achieved superior sensitivity over traditional brightfield techniques. This strategy allows for the capture of all X‐gal precipitates in SABG‐stained samples, revealing diverse X‐gal staining patterns and improved detection sensitivity. Additionally, we demonstrate that reflected light outperforms western blot analysis for the detection and quantification of senescence in mature human adipocytes, as it offers a more accurate representation of SABG activity. This detection strategy enables a more thorough investigation of senescent cell characteristics and specifically a deeper look at the relationship between adipocyte senescence and obesity associated disorders, such as T2D.

## Graphical abstract

Senescence‐associated beta‐galactosidase (SABG) activity was measured using reflected light microscopy by replacing the dichroic mirror with a T80/R20 mirror. SABG activity was manipulated by adjusting the pH, providing both a positive control and a threshold for SABG positivity, enabling objective quantification of senescence. This method is compatible with simultaneous antibody staining.

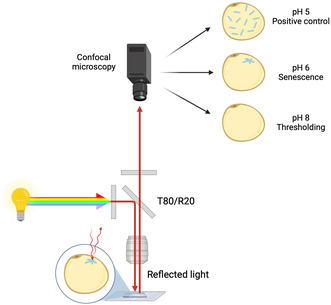

**Abbreviations**
a.u.: Arbitrary Units
ADRB2: beta‐2 adrenergic receptor
ANOVA: Analysis of Variance
BMI: Body Mass Index
FBS: Fetal Bovine Serum
FITC: Fluorescein Isothiocyanate
INS: Insulin
LAMP2: Lysosome‐Associated Membrane Protein 2
PBS: Phosphate Buffered Saline
PFA: Paraformaldehyde
PPAR‐γ: peroxisome proliferator‐activated receptor gamma
R: R Programming Language
RT: Room Temperature
SABG: Senescence‐Associated Beta‐Galactosidase
SD: Standard Deviation
T2D: Type 2 Diabetes
X‐gal: 5‐Bromo‐4‐Chloro‐3‐Indolyl‐Beta‐D‐Galactopyranoside

## 1 INTRODUCTION

Cellular senescence is a complex process that is triggered by an array of stressors such as DNA damage, telomere shortening, or oncogene activation. This process ultimately results in cell‐cycle arrest and an increased resistance to apoptosis through the upregulation of anti‐apoptotic proteins (Wang, 1995; Yosef et al., 2016). Senescence has been associated with enhanced cell survival in situations where it is vital for tissue repair and maintenance (Jun & Lau, 2010; Krizhanovsky et al., 2008). Senescence also plays an important role in development and cancer, where it has been demonstrated that cellular senescence controls embryonic patterning (Muñoz‐Espín et al., 2013; Storer et al., 2013) and cancer cell clearance (Eggert et al., 2016; Feldser & Greider, 2007; Xue et al., 2007), respectively. While senescence serves as a crucial response to various stress stimuli with potential beneficial roles, recent advancements have shed light on another aspect of senescence—the development of a pro‐inflammatory secretory profile that can contribute to local metabolic disruptions, tissue degradation, and even promote tumor progression (Acosta et al., 2013; Coppé et al., 2010; Xu et al., 2015, 2018).

Senescence has been observed in a variety of cells in multiple tissues in association with aging, disease, dysfunction, or cancer. Studies in liver have shown that senescence in hepatic stellar cells can drive cancer progression by disrupting metabolic homeostasis through secreted factors (Li et al., 2020; Yoshimoto et al., 2013). Other studies, which focus on hepatocytes (Aravinthan et al., 2013), or whole liver biopsies (Baboota et al., 2022), demonstrate that senescence in these cells can lead to metabolically associated comorbidities such as non‐alcoholic fatty liver disease or metabolic dysfunction‐associated steatohepatitis. Moreover, the link between Type 2 diabetes (T2D) and senescence has gained a lot of attention the past decade with key findings of associations between senescence and diabetes‐associated complications in pancreatic β‐cells (Aguayo‐Mazzucato et al., 2019; Krishnamurthy et al., 2006; Sone & Kagawa, 2005), hepatocytes (Aravinthan et al., 2013), and adipocytes (Chen et al., 2015; Gustafson et al., 2022; Li et al., 2021; Minamino et al., 2009).

The signaling and protein expression changes of senescent cells are often tissue and cell type specific, however, some changes are considered globally representative of senescent cells. One such biomarker is increased enzymatic activity of beta‐galactosidase at pH 6.0, referred to as senescence‐associated beta‐galactosidase (SABG) (Dimri et al., 1995). Beta‐galactosidase is a tetrameric hydrolase that is expressed in all cells, resides in lysosomes, and is active at acidic pH (lysosomal pH, ~4.5–5.0). Beta‐galactosidase is involved in the lysosomal breakdown of metabolites by catalyzing glycosidic bonds such as galactose and ceramide, but can also cleave synthetic compounds like 5‐bromo‐4‐chloro‐3‐indolyl‐beta‐D‐galactopyranoside (X‐gal) to yield galactose and indole (Horwitz et al., 1964). When this reaction occurs, the released indole homodimerizes and becomes an intensely blue, opaque crystal. This reaction is particularly helpful to visualize senescent cells by incubating samples with X‐gal at pH 6.0 to detect SABG, but not beta‐galactosidase activity which operates at lower pH levels. Traditionally, this blue precipitate has been detected and quantified using brightfield microscopy.

Over time, more senescence hallmarks have been identified, such as increased expression of cyclin‐dependent kinase inhibitors P16, P21 and P53 (Alcorta et al., 1996; Hara et al., 1996; Lin et al., 1998; Okamoto et al., 1994; Serrano et al., 1993, 1997; Wong & Riabowol, 1996). This has led to a more robust classification of senescence where cells should be positive for several senescence markers to be deemed senescent. Nuclear senescent markers, however, such as P16 are usually detected using fluorescence approaches while SABG‐activity is traditionally visualized in brightfield microscopy by scoring blue precipitates. The different nature of these methodologies has limited multi‐modal senescence quantification at a single‐cell level.

The detection of SABG activity in primary mature human adipocytes presents a unique challenge due to their significantly larger size compared to cells in other tissues, with mature adipocytes typically having a diameter of approximately 50–150 microns. Due to the large lipid droplet, lysosomes are more dispersed and the blue precipitates are either scattered into smaller crystals or accumulate to create larger, nest‐like crystal structures (Li et al., 2021). In brightfield microscopy, the small size of the crystals presents a significant challenge: Manual quantification of these minute blue crystals is subjective and likely leads to an underestimate of the true levels of senescence. Moreover, cells scored for SABG activity using brightfield microscopy lack the ability to detect other senescent markers within the same cell. To overcome these limitations, we have developed a confocal microscopy approach that utilizes reflected light to quantify SABG activity by measuring X‐gal precipitates. This approach also introduces positive and negative controls which significantly improve SABG quantification and reliability. Collectively, we describe a strategy that offers a higher resolution of SABG staining, is more sensitive, less subjective and can accommodate immunofluorescence at a single cell level, offering a more nuanced and robust look at senescence.

## 2 METHODS

### 2.1 Human samples

Human surgical biopsies from subcutaneous white adipose tissue from the supra umbilical aspect of the abdominal wall were obtained from 11 patients with obesity undergoing bariatric surgery at Ersta Hospital or Danderyd Hospital in Stockholm, Sweden. Patients were fasted overnight, and fasting blood glucose, insulin, and triglycerides were measured either the morning of surgery (Ersta Hospital) or 2 weeks prior to surgery (Danderyd Hospital). Bloodwork was analyzed at Karolinska Universitetslaboratoriet. Table S1 contains a detailed description of the cohort used for Figure S4. All experiments were performed in accordance with the statutes of the Declaration of Helsinki. The study was approved by the regional ethics committee in Stockholm (Regionala etikprövningsnämnden i Stockholm, 2008/1010 and 2017/1156). Informed written consent was obtained from all participants prior to surgery.

### 2.2 Animals

All experiments involving animals were approved by the regional animal ethics Committee of Northern Stockholm, Sweden (ethical permit no. 4359‐2020). Mice were housed in groups of two to four per cage (GM500 cages, Tecniplast), at approximately 22°C, with ad‐libitum access to water and standard rodent chow diet (Special Diets Services, #801722 CRM (P)), and on a 12 h light/dark cycle. The health status of mice was monitored, and only mice that displayed good general health were used for experiments. C57BL/6N mice were purchased from Janvier Labs. Epididymal adipose tissue from 5‐month‐old males was collected for subsequent adipocyte isolation.

### 2.3 Adipocyte isolation

Human and mouse adipocytes were isolated from white adipose tissue biopsies as previously described (Hagberg et al., 2018) with the exception of omitting BSA from all solutions in the current study. In brief, the tissue sample was mechanically minced, followed by digestion in KrebsRinger‐phosphate buffer (127 mM NaCl, 12.3 mM NaPO_4_, 1.36 mM CaCl_2_, 5.07 mM KCl, 1.27 MgSO_4_, pH 7.4) containing 0.05% collagenase I from Clostridium histolyticum (Sigma, #C0130), 5 mM D‐glucose, 50 μg/mL gentamicin in a 37°C oscillating water bath for 45–60 min. Any remaining undigested tissue was removed by filtration using a 250 μm nylon filter. Floating adipocytes were washed three times with wash buffer containing 5 mM D‐glucose and 50 μg/mL gentamicin in DPBS at pH 7.4. After removing the last round of wash buffer, isolated adipocytes were fixed with fixative buffer (2% PFA and 1% sucrose in PBS) for 20 min on a slow rocker at room temperature (RT), washed with wash buffer and left in PBS for further staining.

Alternatively, cells were transferred to 2 mL tubes, flash frozen in liquid nitrogen and stored at 80°C for protein extraction (see below).

### 2.4 Cell culture and irradiation

After isolation, ~1000 μL of mature adipocytes were placed in basal media (DMEM/F12 medium containing 5 mM glucose, Gibco, Thermo Fisher, #11966025 and #21765029 supplemented with 10% FBS and 1x penicillin–streptomycin). Cells cultured with insulin had basal media supplemented with 100 nM insulin (Sigma‐Aldrich, I‐034) for 7 days. After 7‐day culture, cells were washed in PBS and fixed (2% PFA and 1% sucrose in DPBS) for SABG assay (see below).

For irradiation experiments, freshly isolated adipocytes were placed in basal media and exposed to 5 Gy irradiation using a CIX2 x‐ray cabinet by Xstrahl, administered at a rate of 1.342Gy/min. The adipocytes were subsequently cultured for 5 days to allow for the onset of senescence.

### 2.5 3T3‐L1 adipocyte differentiation and bleomycin treatment

3T3‐L1 cells were plated at a cell density of 20.000 cells/cm^2^. When cells were ~ 70% confluent, differentiation was initiated with adipocyte‐induction media using DMEM/F12 containing 5 mM glucose, supplemented with 10% FBS and 1x penicillin–streptomycin, 66 nM insulin (SigmaAldrich, #I‐034), 10 mM rosiglitazone (Sigma‐Aldrich, #R2408), 0.5 mM dexamethasone (SigmaAldrich, #D4902), 0.5 mM 3‐isobutyl‐1‐methylxanthine (IBMX) (Sigma‐Aldrich, #I5879) for 48 h. Dexamethasone and IBMX were then removed and the cells were allowed to differentiate in 66 nM insulin and 10 mM rosiglitazone for 7 days.

After differentiation, cells were either treated with 25 μg/mL bleomycin (Abcam, #ab142977) or vehicle (100 nM insulin) for 24 h. The cells were then placed in media containing 100 nM insulin to rest for 4 days, after which they were collected using TrypLE (Thermo, #12604021) and fixed for further analysis.

### 2.6 Protein extraction and quantification

Frozen adipocytes were removed from −80°C storage. The thawed adipocytes were mixed at a ratio of 2:1 with SDS buffer (Sigma, #05030‐500ML‐F) (~500 μL adipocytes +250 μL SDS buffer (1%)) and 1:100 Halt™ Protease & Phosphatase Single‐Use Inhibitor Cocktail (100X) (Thermo Scientific, #78442). Tubes were placed on a heating block at 95°C for 5 min. After heating, the tubes were incubated at RT for 15 min on a rocker.

Samples were centrifuged at 14000 rcf for 10 min and the aqueous phase transferred to a new tube. This process was repeated until all lipid and debris were removed. Protein concentration was determined using a Pierce™ BCA Protein Assay (Thermo Scientific, #23227).

### 2.7 Western blot

Ten μg of protein was mixed with 4x Laemmli buffer (BioRad, #1610747) supplemented with 5% 2‐mercaptoethanol (BioRad, #1610710) in a 0.5 mL tube. Samples were heated in a heating block at 95°C for 5 min and centrifuged at 20817 rcf for 1 min.

Running buffer was prepared by mixing 900 mL MQH_2_O with 100 mL 10x TGS stock solution (BioRad, #1610772). Protein samples and 3 μL of Precision Plus Protein Unstained Standards (BioRad, #1610363) were loaded onto a Mini‐PROTEAN TGX Stain‐Free Gel (BioRad, #4568094). Samples were run at 200 V for 40 min using a PowerPacTM Basic (BioRad, #1645050).

After electrophoresis, the gel was activated using a ChemiDocTM MP Imaging System (BioRad, #12003154), using stain‐free technology. A low fluorescence membrane was activated in methanol and filter papers soaked in transfer buffer (Trans‐Blot Turbo RTA Transfer Kit LF PVDF, BioRad, #1704274) before constructing the transfer cassette. Protein transfer was run for 7 min on a mixed molecular weight program using a Trans‐Blot Turbo Transfer System (BioRad, #1704150). The membrane was imaged to ensure complete transfer occurred.

#### 2.7.1 Immunoblotting

The membrane was blocked with EveryBlot Blocking Buffer (BioRad, #12010020) for 10 min on a shaker at RT. To allow for incubation in both beta‐galactosidase antibody (Cell signaling, 1:1000, #271985) and LAMP2 antibody (Atlas antibodies, 1:1000, HPA029100), the membrane was cut at approximately 90 kDa. The two halves were incubated overnight at 4°C on a shaker. Antibodies were diluted in 5% skim milk (Millipore, #70166‐500G). Primary antibody was removed, and the membrane was washed three times for 10 min each with TBS‐T (ChemCruz, #SC‐362311) on a shaker at RT. The membrane was incubated in Anti‐Rabbit IgG Peroxidase secondary antibody (Abcam, 1:5000, #ab206722) diluted in 5% skim milk for 1 h at RT on a shaker. After incubation, the membrane was washed twice for 10 min each with TBST and once for 10 min with PBS on a shaker at RT. SuperSignalTM West Femto Maximum Sensitivity Substrate (Thermo Scientific, #34096) was prepared (5 mL) and the membrane incubated for 5 min on a shaker at RT. The membrane was imaged in the ChemiDocTM MP Imaging System with chemiluminescence and later quantified in ImageLab (BioRad).

#### 2.7.2 Western blot analysis

Following protein transfer, stain‐free imaging of the membrane was carried out and the ImageLab volume tool used to quantify the total protein in each well, adjusting for background. Beta‐galactosidase and LAMP2 chemiluminescent signals were normalized to the total protein of the corresponding well. Beta‐galactosidase levels were normalized to lysosomal content (LAMP2) using chemiluminescence. This normalization was the basis for comparing beta‐galactosidase content, as a function of lysosomal content, across the patient cohort.

### 2.8 X‐gal staining and slide preparations

Freshly isolated or cultured, fixed adipocytes and differentiated 3T3‐L1 cells were analyzed for SABG activity using a Senescence β‐Galactosidase Staining Kit (Cell Signaling, #9860) according to the manufacturer's instructions. The staining solution was adjusted to the indicated incubation pH using either HCl (Sigma, #G7893) or NaOH (Sigma, #72068). Cells were fixed with 2% PFA and 1% sucrose in PBS for 20 min at RT, followed by a 24 h incubation with staining solution, at 37°C (unless otherwise indicated). Cells were washed with PBS, incubated in PBS with 1:500 FITC‐labeled *Lens culinaris* agglutinin (Vector Laboratories, #FL‐1041) for 10 minutes at RT on a slow rocker, washed with PBS three times, filtered through a 200‐micron filter (BD Biosciences, #340644) and plated on slides with ProLong™ Glass Antifade Mountant with NucBlue™ Stain (Invitrogen, #P36983). Slides were cured overnight at RT, coverslipped in glycerol and sealed using nail polish prior to imaging.

X‐gal and antibody staining: Cells retrieved after X‐gal staining were washed in PBS and blocked for 1 h at RT with 5% normal donkey serum (Jackson ImmunoResearch, #017‐000‐121) in PBS, with (P16 and PPARγ_2_) or without (ADRB2) 0.1% Triton X‐100 on a rocker. Cells were incubated with primary antibody (Anti‐CDKN2A/p16INK4a, 1:100, abcam #ab108349; Anti‐ADRB2, 1:100, Santa Cruz Biotechnology #sc‐81577; Anti‐PPARγ_2_, 1:100, Santa Cruz Biotechnology #sc166731) for 1 h at RT on a rocker. Cells were washed in PBS three times and incubated with secondary antibody (Donkey Anti‐Mouse Alexa Fluor™ 555, 1:500, Thermo Fisher #A‐31570; Donkey Anti‐Rabbit Alexa Fluor™ 555, 1:500, Thermo Fisher #A‐31572) for 1 h at RT with 1:500 FITC‐labeled *Lens culinaris* agglutinin (Vector Laboratories, #FL‐1041). Cells were washed and plated as described above.

For control imaging experiments (Figure S6), cells were not stained with X‐gal staining solution while antibodies and preparation procedures were followed as described above.

### 2.9 Microscopy

Cells were imaged on a Leica Stellaris system 5 X (Leica Microsystems). A 20x objective with glycerol‐accommodating immersion oil was used for all experiments. For samples without an antibody stain, a single lane was used for Hoechst, FITC and reflected light. Reflected light was achieved by replacing the dichroic mirror (reflecting excitation light, passing through the emission light) with a T80/R20 mirror (80% transmission, 20% reflection). Reflected light settings were applied to the far‐red channel and the emission spectra for that channel was adjusted to ~630–660 nm to prioritize the reflected light signal. The FITC channel was used for visualizing cell membranes and was used to guide the imaging range to ensure that entire cells were imaged. To avoid quantification of overlapping mature human and mouse adipocytes, dense and clumped cells were excluded from the image analysis. For each sample, a minimum of three separate positions with tiling were imaged. A z‐step increment of 3.1 microns was used and the range for imaging the entire cell was guided by the membrane stain (FITC). Approximately 100 adipocytes were imaged and analyzed from each sample, including pH 8.0 controls, across all channels. Acquired images were stored as raw data files in. lif format.

For differentiated 3T3‐L1 cells, a z‐step increment of 5.0 microns was used and the range for the imaging of cells was guided by the membrane stain (FITC). Approximately 200 cells were imaged and analyzed from each condition.

For samples stained with antibodies, two lanes were used: *Hoechst* and *555 channel* in lane 1, and *FITC* and *reflected light* (647) in lane 2. Splitting was done to minimize fluorescence bleed through.

### 2.10 Image analysis

Raw data files were exported as ImageJ. tif files and loaded into Fiji (National Institute of Health) for analysis. To reduce non‐specific reflected light signal, the LABKIT plugin was used (Arzt et al., 2022). In brief, an image of a representative pH 5.0 sample was captured with z‐step increments of 3.1 microns, covering the full depth of the cells and projected to a maximum image projection. The image was used to train a pixel classifier by distinguishing between X‐gal signal and background. The same pixel classifier and methodology was used for mature human and mouse adipocytes. The pixel classifier for differentiated 3T3‐L1 cells was based on the pH 5.0 sample from 3T3‐L1 cells. Image analysis data was exported as excel files and further analyzed in R or visualized in Prism 9 (GraphPad). Statistical analysis was performed in R and in Prism 9. Statistical significance was considered as *p < 0.05, **p < 0.01, ****p < 0.0001, ns = non‐significant.

### 2.11 SABG scoring

The percentage of SABG positive cells from manual quantification in Figure 3B and Figure S1F are reported as the mean value from two double‐blinded scorers. Manual quantification was done on the same day as confocal microscopy imaging for the same samples. In total, 100 cells were counted by both scorers for each sample. Thresholds (red dotted lines) indicated in Figure 2C,F,I,N, and Figure S2C,F,I,N were calculated from the mean + 3 SD reflected light arbitrary units (a.u.) from sample‐matched controls incubated at pH 8.0, visualized in Figure 2G,H, and Figure S2G,H.

## 3 RESULTS

### 3.1 Enhancing X‐gal detection with reflected light

**FIGURE 1:**
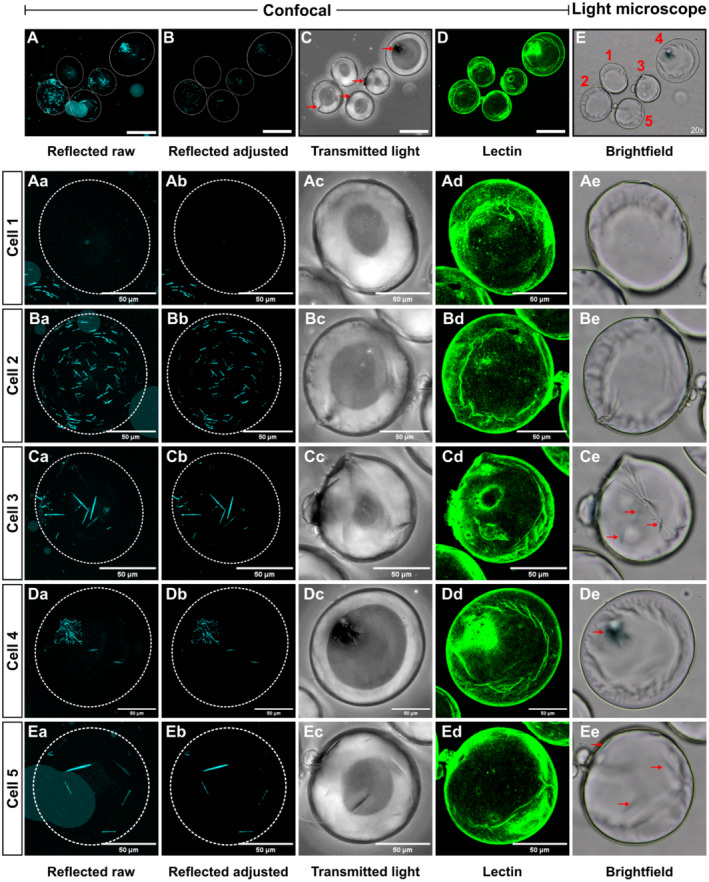
Overview of differences in X‐gal staining observed using confocal reflected light and brightfield microscopy in human adipocytes. Maximum image projection of five mature human primary adipocytes stained with X‐gal, visualized by (A) confocal raw reflected light, (B) confocal reflected light adjusted, (C) confocal transmitted light (dark spots represent opaque X‐gal precipitates, indicated by red arrows) and (D) cell membrane stain lectin. (E) Brightfield image of the same five mature human primary adipocytes. (Aa‐Ee) Digitally zoomed images of cells from (A‐D) showing raw reflected light, reflected light adjusted, transmitted light, lectin and brightfield. Dotted white lines indicate cell perimeter. Red numbers indicate cell numbers. Red arrows highlight blue X‐gal precipitates identified in transmitted light and brightfield microscopy. All images were captured using a 20x objective. White scalebars in A, B, C and D are equivalent to 100 μm.

Building on the established use of X‐gal based SABG staining as a tool for visualizing senescent cells, we investigated the fluorescence detectability of SABG staining in mature human adipocytes using confocal microscopy. A previous study demonstrated that 30 μm thick brain tissue sections stained with X‐gal can be visualized using fluorescence with a peak emission range of ~650–770 nm (Levitsky et al., 2013). In our hands, imaging X‐gal incubated adipocytes with a mean diameter of ~80 μm resulted in a weak fluorescence signal (600–800 nm range), but was markedly improved by capturing the reflected light from an excitation laser (Figure 1A,B); these sources of reflected light partially correspond to the opaque X‐gal crystals, which manifest as dark spots in the transmitted light image (Figure 1C, indicated by arrows).

Biological samples on microscope slides exhibit various sources of reflected light, including dense cell membranes, intracellular structures, cover slip imperfections, and other contaminants which contribute to background noise (Figure 1A). To adjust for background noise, we employed a machine learning‐based pixel classification technique to obtain a more focused X‐gal signal (Figure 1B) (Arzt et al., 2022). To facilitate single‐cell image segmentation, we utilized lectin as a cell membrane stain to delineate the cell perimeter (Figure 1D). With a robust X‐gal signal from the confocal microscope, we evaluated the conventional SABG scoring method, which relies on manual quantification through light‐based brightfield microscopy (Figure 1E), against our reflected light‐fitted confocal technique (herein referred to as confocal reflected light). We observed that primary mature human adipocytes incubated with X‐gal at pH 6.0 exhibit distinct signal phenotypes (Figure 1Aa–Ee). These can be broadly categorized into three types: dispersed (Figure 1Bb), a bundled nest‐like structure (Figure 1Db), or a combination of the two (Figure 1Cb).

When comparing the same cells using confocal reflected light or brightfield microscopy, the nest like structures are easily detected in both methods (Figure 1Db,De). Similarly, cells that are negative using confocal reflected light are also negative in brightfield microscopy (Figure 1Ab,Ae). Cells that exhibit a more dispersed signal profile, however, are more challenging to score in brightfield microscopy compared to confocal reflected light (Figure 1Cb,Ce,Eb,Ee). Interestingly, while Cell 2 shows no X‐gal precipitate in brightfield microscopy (Figure 1Be), it elicits a robust signal when using confocal reflected light (Figure 1Bb). This discrepancy is likely due to the limitations of brightfield microscopy, which captures only singular focal planes, opposed to the sensitivity of confocal microscopy that can image smaller, dispersed crystals throughout the full depth of the cell. Thus, confocal reflected light provides enhanced sensitivity and precision in assessing SABG staining in contrast to the less sensitive and subjective ranking system of traditional brightfield microscopy.

### 3.2 Quantifying SABG activity using reflected light

**FIGURE 2:**
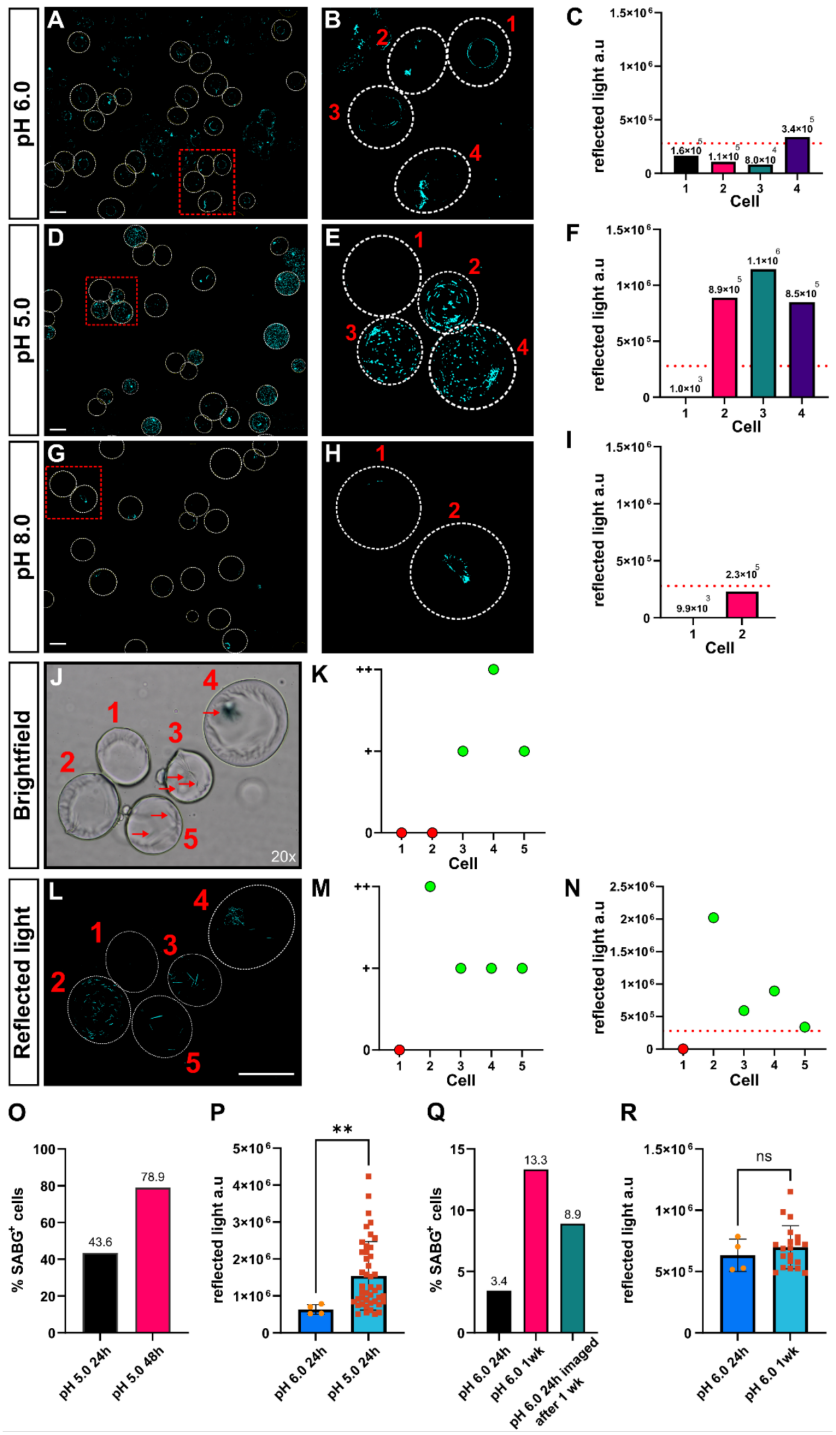
Establishing pH controls for the thresholding of SABG positive human adipocytes. (A) Cells incubated with X‐gal at pH 6.0 to detect SABG activity. (B) Digitally zoomed in image of red box in (A), showing cell perimeters and (C) quantification of confocal reflected light intensity per cell. (D) Cells incubated with X‐gal at pH 5.0 to detect total beta‐galactosidase activity. (E) Digitally zoomed in image of red box in (D), with cell perimeters and (F) reflected light intensities per cell. (G) Cells incubated with X‐gal at pH 8.0 to measure background reflected light for thresholding. (H) Digitally zoomed in image of red box in (G) with cell perimeters and (I) reflected light intensities per cell. Signal at pH 8.0 stems from background reflected light and is the basis for the threshold indicated by the dotted red lines in (C), (F), (I), and (N). (J) Cells from Figure 1 viewed using brightfield microscopy. (K) Scoring of cells in (J) based on blue precipitates indicated by red arrows. (L) Reflected light maximum image projection of cells from Figure 1. (M) Visual scoring of cells in (L) based on blue signal. (N) Scoring of cells based on the threshold established from cells in (G), with reflected light intensities per cell. (O) Percentage of SABG positive cells incubated with X‐gal at pH 5.0 for 24 or 48 h. (P) Subset of SABG positive cells showing reflected light intensity per cell in samples that were incubated at pH 6.0 or pH 5.0 for 24 h. Each symbol represents one cell. (Q) Percentage of SABG positive cells stained at pH 6.0 for 24 h or 1 week. ‘pH 6.0 24h’ and ‘pH 6.0 24h imaged after 1 week’ represent measurements from the same slide, with the ‘late’ measurement taken 1 week after the initial ‘24 h’ assessment. (R) Subset of SABG positive cells showing reflected light intensity per cell in samples that were incubated at pH 6.0 for 24 h or 1 week. Each symbol represents one cell. Dotted white lines indicate cell perimeter. Dotted red lines in (C), (F), (I) and (N) correspond to the threshold for SABG positive cells. Red, dotted squares indicate the area of digital zoom. Numbers above graphs in (C), (F) and (I) correspond to the reflected light intensity for the indicated cell. The numbers above graph bars in (O) and (Q) indicate the percentage of SABG positive cells per condition. ** = *p* < 0.01 unpaired two‐tailed Mann–Whitney test, ns = nonsignificant. White scalebars in A, D, G and L are equivalent to 100 μm.

Confocal reflected light introduces a quantifiable metric by measuring the pixel values of reflected light from the opaque X‐gal precipitates. To ensure that the reflected light signal in X‐gal treated samples stems from the precipitated crystals, and not from highly folded membranes or contaminants, samples were incubated at varying pH levels. At pH 6.0, the reflected light signal should be directly proportional to the SABG activity in the sample (Figure 2A,B) (Dimri et al., 1995). By lowering the pH to a more lysosomal working‐range, pH 5.0, a larger portion of the beta‐galactosidase in the cell should participate in the breakdown of X‐gal and produce a higher reflected light signal, as seen in Figure 2D,E. Similarly, increasing the pH to 8.0 diminishes beta‐galactosidase activity and globally decreases the reflected light signal in the sample (Figure 2G,H). Signal that is present at pH 8.0 thus stems from background reflected light and can be used to create a threshold for defining SABG positive from SABG negative cells. Lectin staining was leveraged to segment individual cells to calculate reflected light pixel values for each cell (dotted white circles, Figure 2B,C,E,F,H,I). While most cells exhibit a near‐zero reflected light signal at pH 8.0, some cells elicit signals between 0 and 250 × 10^3^ reflected light a.u. (Figure 2H,I). The threshold for SABG positive cells is derived from the mean plus three standard deviations of the measurements obtained from cells in the pH 8.0 X‐gal treated sample (Figure 2g), as indicated by the dotted line in Figure 2C,F,I,N.

When comparing the same cells in brightfield microscopy and confocal reflected light (Figure 2J,L), cells can be scored visually (Figure 2K,M), or by using pixel values (Figure 2N). Using confocal reflected light, with sample‐matched pH 8.0 controls for thresholding (Figure 2N), more SABG positive cells were detected compared to brightfield microscopy (Figure 2K,M). Given the pronounced variability in size, oiliness, color, and fragility of primary mature human adipocytes between patients, a pH 8.0 threshold for each unique patient sample is recommended.

### 3.3 X‐gal signal is time‐dependent

When the pH is reduced (pH 5.0) to force a strong signal in the X‐gal incubated samples, not all cells exhibit a strong reflected light signal (Cell 1, Figure 2E,F). To investigate this further, the same sample was incubated for twice the recommended incubation time (48 h instead of 24 h). A longer incubation time, at same pH as the control, substantially increased the percentage of X‐gal positive cells (43.6% vs. 78.9%, Figure 2O). Moreover, the signal from pH 5.0‐incubated samples emitted a significantly stronger signal per cell (**, Mann–Whitney, *p* = 0.0053) compared to pH 6.0 incubated samples (Figure 2P). This increased signal per cell at pH 5.0 is most likely attributed to the basal activity of beta‐galactosidase at the lower pH, leading to increased X‐gal cleavage, rather than being a direct reflection of SABG activity. Interestingly, X‐gal signal also increased at pH 6.0 if samples were incubated for longer periods of time (24 h vs. 1 week), or if the same slide was imaged at different time‐points (24 h vs. 1 week, Figure 2Q). Although the percentage of SABG positive cells increases with longer incubations, the average signal intensity per SABG positive cell remains consistent (Figure 2R).

### 3.4 X‐gal detection in mouse adipocytes

To investigate if X‐gal detection using reflected light is compatible with other primary cells, mature mouse adipocytes isolated from the epididymal depot of 5‐month‐old chow‐fed mice were incubated with X‐gal at pH 6.0 and imaged with both brightfield and confocal microscopy (Figure S1A–E). Similar to the findings in human adipocytes, both X‐gal crystals and background reflected light were readily detected (Figure S1A). Machine learning‐based pixel classification further refined the X‐gal crystal signal, and lectin staining enabled single‐cell classification (Figure S1B,D). The general background noise, however, was more pronounced compared to human data, even after pixel classification (Figure 1B (human), Figure S1B (mouse)). Cell 1 displayed a reflected light pattern similar to the scattered phenotype observed when human adipocytes were cultured at pH 5.0 (Figure 2E (human), Figure S1Aa–Ae (mouse)), while cells 2, 3, and 4 showed patches of reflected light (Figure S1Ba‐bd,Ca‐Cd,Da‐Dd). None of the four cells were considered positive for blue X‐gal crystals in brightfield microscopy (Figure S1E,Ae,Be,Ce,De).

To quantify the reflected light signal in mature mouse adipocytes, the signal intensity per cell was measured using the same approach as with human data. Following incubation at pH 6.0, 8.9% of mouse adipocytes were SABG positive (Figure S2A–C,O). These positive cells exhibited higher signal intensities per cell compared to human adipocytes incubated at the same pH (Figure 2C,P [human], Figure S2C,P [mouse]). When incubated at pH 5.0, mouse adipocytes displayed both higher signal intensity per cell and a greater fraction of positive cells after 24 h compared to those incubated at pH 6.0 (Figure S2D–F,O,P). Additionally, mouse adipocytes incubated at pH 5.0 showed higher intensities per cell and a greater fraction of positive cells compared to human adipocytes under the same conditions (Figure 2O,P [human], Figure S2O,P [mouse]). This trend of higher signal intensity per cell in mouse adipocytes was also observed at pH 8.0, where signal intensities between 0 and 500 × 10^3^ reflected light a.u. were common. The threshold (mean plus three standard deviations) for detecting SABG positive cells in mouse adipocytes was roughly three times higher than that in human adipocytes (0.95 × 10^3^ reflected light a.u in mouse versus 0.28 × 10^3^ reflected light a.u in human).

Despite being the same cell type (primary mature adipocytes), differences in species, tissue origin, and prior in vivo conditions likely contribute to variations in background reflected light signal and X‐gal crystal formation, resulting in different signal intensities. Other factors, such as metabolic and lysosomal activity, could also play a role. Although none of the four cells were considered positive in brightfield microscopy (Figure S2J,K), subjective scoring of reflected light may also be misleading (Figure S2L,M) unless a pH 8.0 threshold is applied (Figure S2N). Therefore, it is crucial to use a pH 8.0 control from the same sample to accurately distinguish background from X‐gal signal, and to establish the threshold for what is considered SABG positive.

### 3.5 X‐gal detection in differentiated 3T3‐L1 cells

To evaluate the applicability of X‐gal detection using reflected light in cell lines, and its sensitivity to detect increases in senescence, 3T3‐L1 cells were differentiated into an adipocyte lineage and subsequently treated with bleomycin. The 3T3‐L1 cell line is commonly used for adipocyte studies due to its well‐characterized differentiation process and similarity to primary adipocytes, in terms of lipid metabolism and gene expression. Previous studies have shown that 3T3‐L1 cells have very low levels of senescence, before and after differentiation into an adipocyte lineage (de Oliveira et al., 2024; Zoico et al., 2019). Although differentiated 3T3‐L1 cells are often used for the study of adipocyte biology, there are marked phenotypic differences from primary mature adipocytes, such as cell size and lipid droplet formation (Figure 1Aa–Ee, Figure S3Aa–Dc). Despite these differences, cells were processed similarly to mature human adipocytes, namely stained with lectin for segmentation and subsequently quantified for reflected light.

Similar to primary mature mouse adipocytes, the majority of adipocyte differentiated 3T3‐L1 cells incubated at pH 5.0 display a robust X‐gal signal and little to no signal when incubated in pH 8.0 (Figures S2O and S3Aa‐Ac,Db‐Dc,E). Compared to control untreated cells, bleomycin treated cells (25 μg/mL 24 h, left to rest for 4 days) demonstrated a robust increase in senescence at pH 6.0 (Figure S3Ba‐Bc,Ca‐Cc,E). While the fraction of SABG positive cells increased with bleomycin treatment, the signal intensity per cell was not significantly higher (ns, one‐way ANOVA with Tukey's post hoc, *p* = 0.9675) compared to SABG positive control cells (Figure S3F). SABG positive cells from both control and bleomycin treatment were significantly lower in intensity per cell compared to pH 5.0 incubated cells (****, one‐way ANOVA, *p* < 0.0001), suggesting that the senescence increase in bleomycin treated cells only partially increases SABG activity without fully activating all lysosomes at pH 6.0, as seen in cells incubated at pH 5.0 (Figure S3Ab‐Ac,Cb‐Cc,F).

### 3.6 Comparing multiple SABG scoring methods

SABG activity was compared across a diverse patient cohort using common SABG‐scoring methods (western blot, brightfield microscopy) against our newly developed confocal reflected light method. We included 8 patients with a mean age of 49 years and mean BMI of 35. Further details on patient characteristics are found in Table S1. SABG levels in adipocytes from 8 patients were compared across three different methods: protein levels from total protein in western blot (Figure S4A,B), confocal reflected light (Figure S4E), and manual quantification using brightfield microscopy (Figure S4F). Beta‐galactosidase levels varied substantially between patients when using western blot and normalizing to total protein (Figure S4A,B). As antibody staining cannot discriminate between beta‐galactosidase and SABG, we controlled for beta‐galactosidase variance due to potential differences in lysosomal content, by normalizing to a common lysosomal marker, lysosome‐associated membrane protein 2 (LAMP2). In our patient cohort, LAMP2 expression varied considerably (Figure S4A,C). When normalizing beta‐galactosidase levels to LAMP2, the variance was reduced (fold change range 1.0–6.0 in nonnormalized and 1.0–2.7 in normalized, Figure S4B,D). Notably, the relative levels of beta‐galactosidase expression among patients shifted when accounting for differences in lysosomal content, particularly in those with high levels of both beta‐galactosidase and LAMP2 (Figure S4A,B,D, patients #2, #3 and #8).

Using the confocal reflected light technique, approximately 100 cells were imaged at full depth (3.1 μm increments to cover the full depth of the cell, see methods for more details) at pH 6.0 for SABG activity, and 100 cells imaged at pH 8.0 for thresholding, for each patient sample. Five out of 8 patients had SABG positive cells (Figure S4E). Using brightfield microscopy, where 100 cells were scored, only one out of eight patients were SABG positive (Figure S4F). The highest scored SABG positive sample using confocal reflected light was notably the only sample with SABG positive cells scored using brightfield microscopy (Figure S4E,F, patient #3). Notably, patient #3, who scored highest for SABG positive cells in confocal reflected light and brightfield microscopy, exhibited only moderate beta‐galactosidase expression in western blot, even after normalization to lysosome content (Figure S4B,D). Interestingly, patient #5, who consistently displayed the lowest beta‐galactosidase protein expression, with and without normalization to lysosomal content (Figure S4B,D), was observed to have 3.8% SABG positive cells by confocal reflected light scoring (Figure S4E). Thus, inferring SABG activity using western blot by detecting beta‐galactosidase is less informative than microscopy approaches. This is perhaps not surprising given that the antibody detects all beta‐galactosidase, including non‐SABG, whereas microscopy approaches only detect by‐products resulting from SABG activity.

### 3.7 Reflected light is a more comprehensive method for the detection of senescence

**FIGURE 3:**
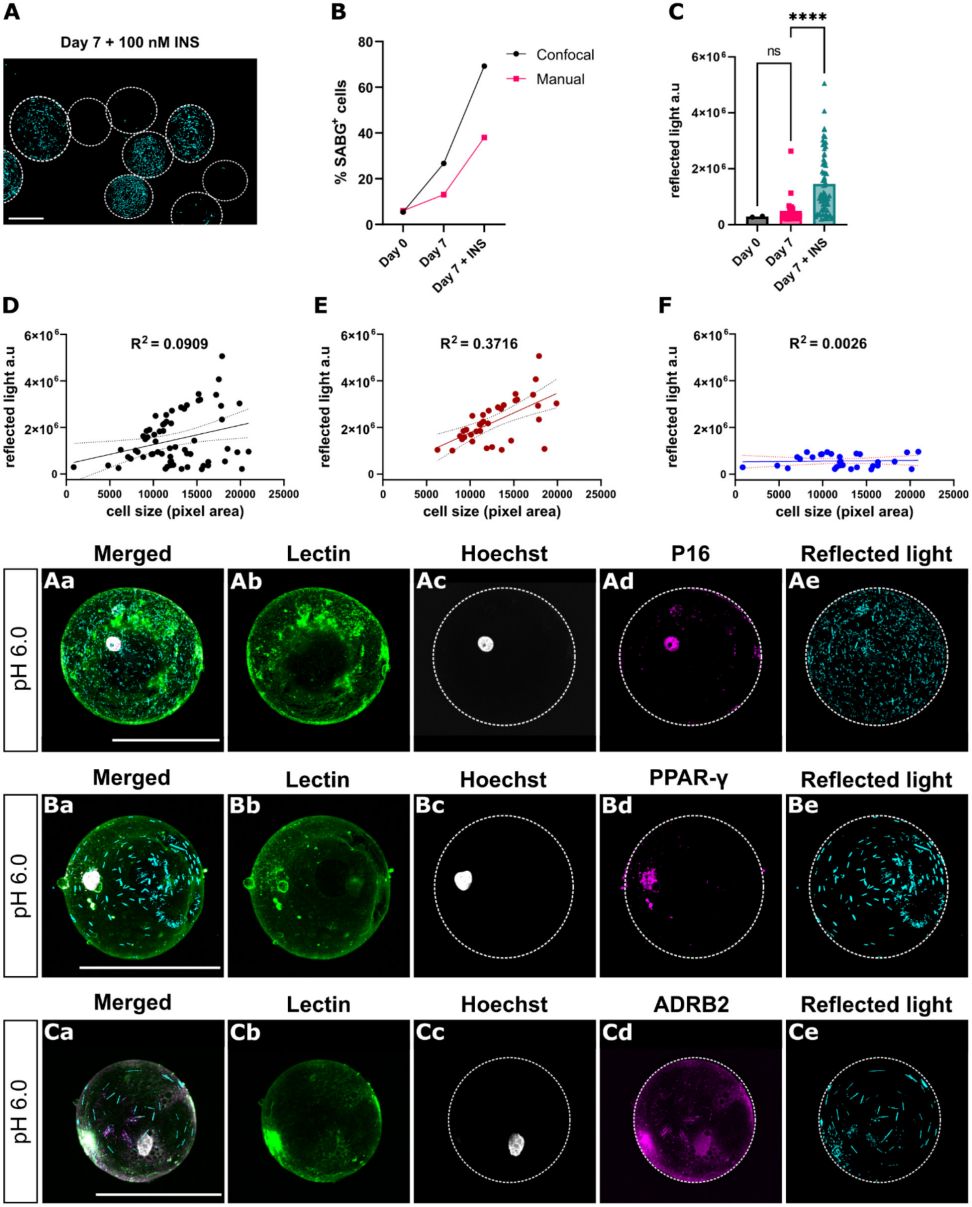
Characterizing SABG positive human adipocytes using confocal microscopy. (A) Representative image of reflected light maximum image projection from mature primary human adipocytes cultured for 7 days with 100 nM insulin. (B) Percentage SABG positive adipocytes at day 0, 7 day culture or 7 day culture with 100 nM insulin, scored using both confocal reflected light and brightfield microscopy. (C) Subset of SABG positive adipocytes with reflected light intensities from (B), measured using confocal reflected light. Each symbol represents one cell. (D) Subset of SABG positive adipocytes that were cultured for 7 days with 100 nM insulin mapped for size and reflected light intensity. Each dot represents one cell. (K) Subset of cells (>1 × 10^6^ reflected light a.u.) from (D). Each dot represents one cell. (L) Subset of cells (<1 × 10^6^ reflected light a.u.) from (D). Each dot represents one cell. (Aa‐Ae) Representative images of primary mature human adipocytes cultured for 7 days with 100 nM insulin, incubated with X‐gal at pH 6.0 for 24 h, stained and visualized for lectin, Hoechst, protein‐of‐interest and reflected light. Nuclear senescence marker P16. (Ba‐Be) Adipocyte nuclear marker PPARγ. (Ca‐Ce) Cell‐surface receptor ADRB2. Dotted white lines indicate cell perimeter. **** = *p* < 0.0001 Kruskal‐Wallis test with Dunn's post hoc test, ns = non‐significant. All images were taken with a 20× objective. White scalebars in A, Aa, Ba and Ca are equivalent to 100 μm. INS = insulin.

Since manual quantification using brightfield microscopy only detected one out of eight patients to contain SABG positive cells, we investigated if this method consistently underestimates SABG activity compared to the confocal reflected light approach. Senescence was induced in primary mature human adipocytes using two separate methods: culturing cells with high concentrations of insulin (100 nM) for 7 days (Q. Li et al., 2021) (Figure 3A) and treating cells with clinically relevant concentrations of irradiation (Figure S5). Irradiation treatment led to an increase in the percentage of SABG positive cells (3.2%) compared to control (1.1%) (Figure S5Aa,Ba,D). While this increase was notable, and clinically relevant as previously reported studies have reported that even a small number of senescent cells could impact tissue function (Kim et al., 2020; Xu et al., 2018), it was less pronounced than the senescence induced by high insulin concentrations. Therefore, to achieve a larger range of senescence for quantification purposes, we primarily used the insulin‐induced approach.

Imaging the same slides using both quantification methods showed that confocal reflected light consistently reports higher levels of SABG compared to brightfield in samples treated with ± insulin for 7 days (Figure 3B). The underestimation from brightfield microscopy may be due to difficulties identifying the smaller, scattered crystals found in these samples (Figure 3A), which resembles Cell 2 in Figure 1Ba,BE.

Using lectin staining to segment individual cells, the intensity per cell under insulin‐induced senescent conditions was significantly higher than in non‐induced conditions (****, Kruskal–Wallis with Dunn's post hoc, *p* < 0.0001) (Figure 3C), similar to intensities observed in adipocytes stained with X‐gal at pH 5.0 (Figure 2D,E,F). The intensity per cell in SABG positive cells from noninduced conditions (Day 7) was not significantly different from SABG positive cells at Day 0 (ns, Kruskal–Wallis with Dunn's post hoc, *p* > 0.9999) (Figure 3C).

Another benefit of confocal microscopy is that it allows for size measurements, reported as either pixels or microns. Using this information, two distinct cell populations from the SABG positive cells in Figure 3C (Day 7 + INS) were identified (Figure 3D) and separated into two groups: The first group consisted of cells with higher intensities (>1 × 10^6^ reflected light a.u./cell), and the second group with lower intensities (<1 × 10^6^ reflected light a.u./cell). Single‐cell intensity, and cell size information, allowed for the investigation of size‐intensity trends in the dataset: In cells classified as having a high SABG signal intensity, cell size scaled with reflected light intensity (Figure 3e). In comparison, SABG positive cells with lower reflected light intensity, showed no correlation between cell size and reflected light intensity (Figure 3F). The variation in reflected light intensity, and scaling with cell size, could indicate differences in lysosomal content and distribution among the cells.

The ability to measure fluorescence from various channels is another benefit of using confocal microscopy to detect reflected light in X‐gal stained samples. Lectin and Hoechst are routinely used to stain adipocyte membranes and nuclei, respectively (Hagberg et al., 2018; Li et al., 2021). We show that these stains can be easily integrated with X‐gal staining in adipocytes (Figures 1C and 3Ab,Ac,Bb,Bc,Cb,Cc).

Building on this integration with dyes, the capabilities of our multimodal technique were expanded by integrating antibody staining to detect additional senescent markers alongside X‐gal staining. Primary human adipocytes were treated with insulin to induce senescence, followed by staining for X‐gal at pH 6.0 and conventional antibody staining. Cells with strong reflected light intensity were positive for P16 (Figure 3Aa–Ae). Important to note, P16 staining following X‐gal staining, at pH 6.0, was not notably different than P16 staining alone (using a conventional antibody staining protocol) (Figure S6Aa–Ae).

To further illustrate the versatility of our method, X‐gal was paired with antibody staining for an additional nuclear marker or a cell surface receptor. Clear detection of the adipocyte nuclear marker PPAR‐γ (Figure 3Ba‐Be and Figure S6Ba‐Be) and the membrane‐bound protein beta‐2 adrenergic receptor (ADRB2) (Figure 3Ca‐Ce and Figure S6Ca–Ce) could be seen. Together, these findings highlight the multimodality of confocal reflected light imaging to accurately determine senescent cell levels, simultaneously with other senescent markers, at a single cell resolution. This approach offers a powerful toolkit for a more comprehensive characterization of cellular senescence, enabling simultaneous X‐gal detection through confocal reflected light and protein detection through immunofluorescence.

## 4 DISCUSSION

Cellular senescence is a well‐established biological process involved in aging, tissue repair, and disease pathogenesis. In metabolic disorders, such as obesity and T2D, cellular senescence is notably increased in adipose tissue, liver, and pancreas. In this study, we used primary cells from human adipose tissue to demonstrate a novel approach that utilizes reflected light to detect X‐gal staining, offering greater sensitivity than conventional brightfield microscopy SABG scoring. Furthermore, this method can be combined with immunofluorescence for the detection of multiple senescent markers, at a single‐cell level.

When assessing SABG activity within the same cohort using conventional methods, our newly developed technique emerges as a highly sensitive, informative, and comprehensive alternative approach for determining adipocyte senescence. Prior investigations have indicated that X‐gal precipitates can be easily detected solely through fluorescence (Levitsky et al., 2013; Matei et al., 2006). We did not observe a robust fluorescent signal stemming from X‐gal precipitates in primary mature human adipocyte samples. By changing the dichroic filter; however, to a T80/R20 mirror (20% reflected light from excitation laser) during acquisition, it is possible to obtain a strong and detectable signal. This signal is directly proportional to SABG activity when incubated at pH 6.0. By manipulating pH levels during X‐gal treatment it is possible to differentiate between beta‐galactosidase and SABG activity and effectively control for background reflected light.

When incubating samples with X‐gal at pH 6.0 for longer than the recommended time, we observed higher levels (3.4%/24 h vs. 13.3%/1 week) of SABG positive cells compared to 24 h incubations (Figure 2o). The intensity per cell was not significantly different from controls, suggesting that low levels of beta‐galactosidase, active at pH 6.0, accumulate signal over time. When incubating samples with X‐gal at pH 5.0 for 48 h, there was a marked increase in the percentage of SABG positive cells compared to the 24 h condition, indicating that some cells may require longer incubations to accumulate X‐gal precipitates (Figure 2Q). These experiments highlight the need to use the same incubation time when comparing SABG staining across sample cohorts and demonstrate that the kinetics of X‐gal precipitate accumulation are not fully understood and warrant further investigation.

The findings in this study underscore the need for using sample‐matched pH 8.0 controls when quantifying SABG positive cells. The variability in background reflected light signal and X‐gal crystal formation between species, as seen in mature human and mouse adipocytes, suggests that there might be biological differences driving these variations. Additionally, the size difference in differentiated 3T3‐L1 cells compared to mature adipocytes indicates that smaller cells, which likely have fewer lysosomes and less beta‐galactosidase per cell, may exhibit different background reflected light and SABG intensities. Employing an objective method for senescence scoring, such as the reflected light method described here, is thus essential for ensuring consistent and reliable senescence quantification across various cell types and experimental conditions.

Confocal reflected light provides a more accurate representation of SABG levels in biologically diverse samples compared to western blot, for three key reasons: (i) Western blot detects the presence of beta‐galactosidase, while reflected light directly measures enzymatic activity by detecting a by‐product resulting from X‐gal cleavage. (ii) Antibodies used for western blot cannot differentiate between beta‐galactosidase and SABG. Previous studies have reported that senescent cells increase their lysosomal and beta‐galactosidase content and activity (Kurz et al., 2000). This is an issue, however, when comparing diverse subjects with variations in basal beta‐galactosidase and lysosomal content, where it is possible for individuals to express high levels of beta‐galactosidase and lysosomal content but show no signs of SABG activity (patient #2, Figure S1B,C,E). (iii) Lastly, western blot typically measures protein levels from a population of cells. This reduced resolution makes it challenging to discern cellular heterogeneity and difficult to determine whether observed changes in protein expressions are due to a small, highly active population of cells, or minor increases distributed across the cell population. Taken together, the significant variations in beta‐galactosidase levels and total lysosomal content, as measured by western blot in our patient cohort, poses significant challenges when relating western blot beta‐galactosidase data to SABG activity.

Confocal reflected light also demonstrates distinct advantages over manual quantification by brightfield microscopy: It is less subjective, reports a greater dynamic range, is more sensitive and allows for coupling with immunofluorescence to simultaneously detect additional senescent markers. By incorporating pH 8.0 controls in the workflow, it is possible to account for background reflected light, eliminating the need for subjective determination of a cell's senescent status. Additionally, using a confocal microscope to capture all precipitates from the X‐gal, and exact size measurements of cells, it is possible to identify distinct senescent phenotypes, and potentially identify multiple subpopulations of senescent cells.

Recently, multiple fluorescence‐based tools for visualizing beta‐galactosidase have been developed (Doura et al., 2016; Lee et al., 2014; Zhao et al., 2023), including significant progress in visualizing beta‐galactosidase in vivo (Hu et al., 2023; Wang et al., 2019). While these tools provide innovative methods for the visualization of beta‐galactosidase, it is important to recognize the inherent complexities in differentiating general beta‐galactosidase activity from SABG activity. An essential component in this differentiation is the control of the pH levels, which is necessary for the accurate identification of SABG (Dimri et al., 1995; Itahana et al., 2013). Tools utilizing beta‐galactosidase trackers could potentially track senescent cells due to an increase in lysosomal content and beta‐galactosidase during senescence (Lee et al., 2006; Rovira et al., 2022; Tan & Finkel, 2023); however, we demonstrate in our human patient cohort that beta‐galactosidase levels, lysosomal content, and SABG are not correlated.

Taken together, this novel and multifaceted approach to characterizing senescence at a single cell level, with the ability to also measure proteins by immunofluorescence, allows for a deeper understanding of cellular senescence. Given the number of different cell types reported to senesce, and the variety of senescent triggers—from oxidative stress and DNA damage to mitochondrial dysfunction and oncogene activation—defining methodologies that allow for a more nuanced evaluation of the senescent state in all sample types are much needed.

## AUTHOR CONTRIBUTIONS

All authors participated in the analysis and interpretation of the data. Benjamin Dedic and Andrea Mosqueda Solís operated and fine‐tuned reflected light signal. Benjamin Dedic, Leo Westerberg and Kirsty L. Spalding designed the experiments. Benjamin Dedic and Leo Westerberg performed the experiments. Erik Näslund and Anders Thorell recruited the human participants. Kyle D. Dumont and Jorge L. Ruas provided mouse tissues. Benjamin Dedic operated the reflected light microscope. Leo Westerberg performed the western blot experiments. Benjamin Dedic and Leo Westerberg wrote the first version of the manuscript, all authors contributed to, and approved the final version.

## FUNDING INFORMATION

This study was supported by grants to JLR from the Novo Nordisk Foundation (NNF21OC0070149), AT from The Erling‐Persson Foundation (140609), and KLS from the Swedish Research Council (2022‐01236), the Strategic Research Program for Diabetes at Karolinska Institutet (C5471162), the Novo Nordisk Foundation (C5475033), Vallee Foundation Scholar Award (C5471234), Mark Foundation Aspire Grant (C5477023), The Swedish Cancer Society (22 2420), the Strategic Research Program for Stem cells and Regeneration at Karolinska Institutet (C5472022) and Knut and Alice Wallenberg Foundation (2020.0118; 2023.0536).

## CONFLICT OF INTEREST STATEMENT

The authors declare no conflict of interest.

## Supporting information


**Table S1.** Patient cohort characteristics for experiments in Figure S1. The cohort consisted of eight people in total. Data are reported as mean and range.
**Figure S1**. Overview of differences in X‐gal staining observed using confocal reflected light and brightfield microscopy in mouse adipocytes. Maximum image projection of four mature mouse primary adipocytes stained with X‐gal, visualized by (A) confocal raw reflected light, (B) confocal reflected light adjusted, (C) confocal transmitted light and (D) cell membrane stain lectin. (E) Brightfield image of the same four mature mouse primary adipocytes. (Aa–Ee) Digitally zoomed images of cells from (A–D) showing raw reflected light, reflected light adjusted, transmitted light, lectin and brightfield. Dotted white lines indicate cell perimeter. Red numbers indicate cell numbers. All images were captured using a 20x objective. White scalebars in A, B, C and D are equivalent to 100 μm, scalebars in Aa‐Dd are equivalent to 50 μm.
Figure S2. Establishing pH controls for the thresholding of SABG positive mouse adipocytes. (A) Cells incubated with X‐gal at pH 6.0 to detect SABG activity. (B) Digitally zoomed in image of red box in (A), showing cell perimeters and (C) quantification of confocal reflected light intensity per cell. (D) Cells incubated with X‐gal at pH 5.0 to detect total beta‐galactosidase activity. (E) Digitally zoomed in image of red box in (D), with cell perimeters and (F) reflected light intensities per cell. (G) Cells incubated with X‐gal at pH 8.0 to measure background reflected light for thresholding. (H) Digitally zoomed in image of red box in (G), with cell perimeters and (I) reflected light intensities per cell. Signal at pH 8.0 stems from background reflected light and is the basis for the threshold indicated by the dotted red lines in (C), (F), (I), and (N). (J) Cells from Figure S1 viewed using brightfield microscopy. (K) Scoring of cells in (J) based on blue precipitates. (L) Reflected light maximum image projection of cells from Figure S1. (M) Visual scoring of cells in (L) based on reflected light signal. (N) Scoring of cells based on the threshold established from cells in (G), with reflected light intensities per cell. (O) Percentage of SABG positive cells incubated with X‐gal at pH 5.0 for 24 h or 48 h. (P) Subset of SABG positive cells showing reflected light intensity per cell in samples that were incubated at pH 6.0 or pH 5.0 for 24 h. Each symbol represents one cell. Dotted white lines indicate cell perimeter. Dotted red lines in (C), (F), (I) and (N) correspond to the threshold for SABG positive cells. Red, dotted squares indicate the area of digital zoom. Numbers above graphs in (C), (F) and (I) correspond to the reflected light intensity for the indicated cell. The numbers above graph bars in (O) indicate the percentage of SABG positive cells per condition. ** = *p* < 0.01 unpaired two‐tailed Mann–Whitney test. White scalebars in A, D, G and L are equivalent to 100 μm.
Figure S3. Overview of differentiated 3 T3‐L1 cells incubated in pH controls and bleomycin treatment. Reflected raw, reflected adjusted and lectin images of cells incubated in pH 5.0 (Aa‐Ac), control pH 6.0 (Ba‐Bc), bleomycin pH 6.0 (Ca‐Cc) and negative control pH 8.0 (Da‐Dc). (E) Percentage of SABG positive cells after control (pH 6.0), bleomycin treatment (pH 6.0) and pH 5.0. (F) Subset of SABG positive cells showing reflected light intensity per cell in samples from (E). Each symbol represents one cell. The numbers above graph bars in (E) indicate the percentage of SABG positive cells per condition. (Ac, Bc, Cc and Dc) are digital magnifications of the respective red boxes. **** = *p* < 0.0001 one‐way ANOVA with Tukey’s post‐hoc. ns = non‐significant. White scalebars in Aa‐Dc are equivalent to 50 μm.
Figure S4. Comparing multiple SABG scoring techniques in human adipocytes. (A) Western blot image of bands corresponding to LAMP2 (~130 kDa), beta‐galactosidase (~65 kDa) and total protein. (B) Western blot densitometry of beta‐galactosidase values. Data is normalized to total protein and visualized as fold change from the lowest value. (C) Western blot densitometry of LAMP2 values. Data is normalized to total protein and visualized as fold change from the lowest value. (D) Densitometry of beta‐galactosidase values normalized to LAMP2. (E) Percentage of SABG positive cells scored using confocal reflected light. (F) Percentage of SABG positive cells scored using brightfield manual quantification. Unique patients are indicated with the same # throughout the figure.
Figure S5. SABG in 5 Gy irradiated mature human adipocytes. Cells were exposed to radiation over 5 min, and then left to rest for 4 days. Reflected light and membrane images of control (Aa‐Ab), irradiated pH 6.0 (Ba‐Bb) and irradiated pH 8.0 (Ca‐Cb) adipocytes. (D) Percentage of SABG positive cells after control and irradiation treatment. Dotted white lines indicate cell perimeter. The numbers above graph bars in (D) indicate the percentage of SABG positive cells per condition. White scalebars in Aa‐Cb are equivalent to 50 μm.
Figure S6. Antibody staining at standard pH (~7.4) without X‐gal staining. Representative images of primary mature human adipocytes cultured for 7 days with 100 nM insulin, stained and visualized for lectin, Hoechst, protein‐of‐interest and reflected light. (Aa‐Ae) Nuclear senescence marker P16. (Ba‐Be) Adipocyte nuclear marker PPAR‐γ. (Ca‐Ce) Cell‐surface receptor ADRB2. Dotted white lines indicate the cell perimeter. All images were taken with a 20x objective. White scalebars in Aa, Ba and Ca are equivalent to 100 μm.

## Data Availability

Data available upon request from the authors. Data citation: The paper contains no omics and/or GWAS datasets.
