# Supplementary material for: Senescence detection using reflected light
Source: Aging Cell. 2024 Aug 5;23(11):e14295. doi: 10.1111/acel.14295 (PMC11561700; doi:10.1111/acel.14295)

Supplementary Table 1

Characteristics	N = 8
Sex (M/F)	6/2
Patient Age	49,5 (28-65)
BMI	35,28 (23,2-42,8)
Weight (kg)	103,95 (73,6-141,8)
Fat %	35,8 (18,6-47,6)
Glucose (mmol/L)	5,65 (5,1-10,1)
Insulin (µIU/L)	12 (8,5-42)
Triglycerides (mmol/L)	1,03 (0,7-2,1)

Supplementary Figure 1

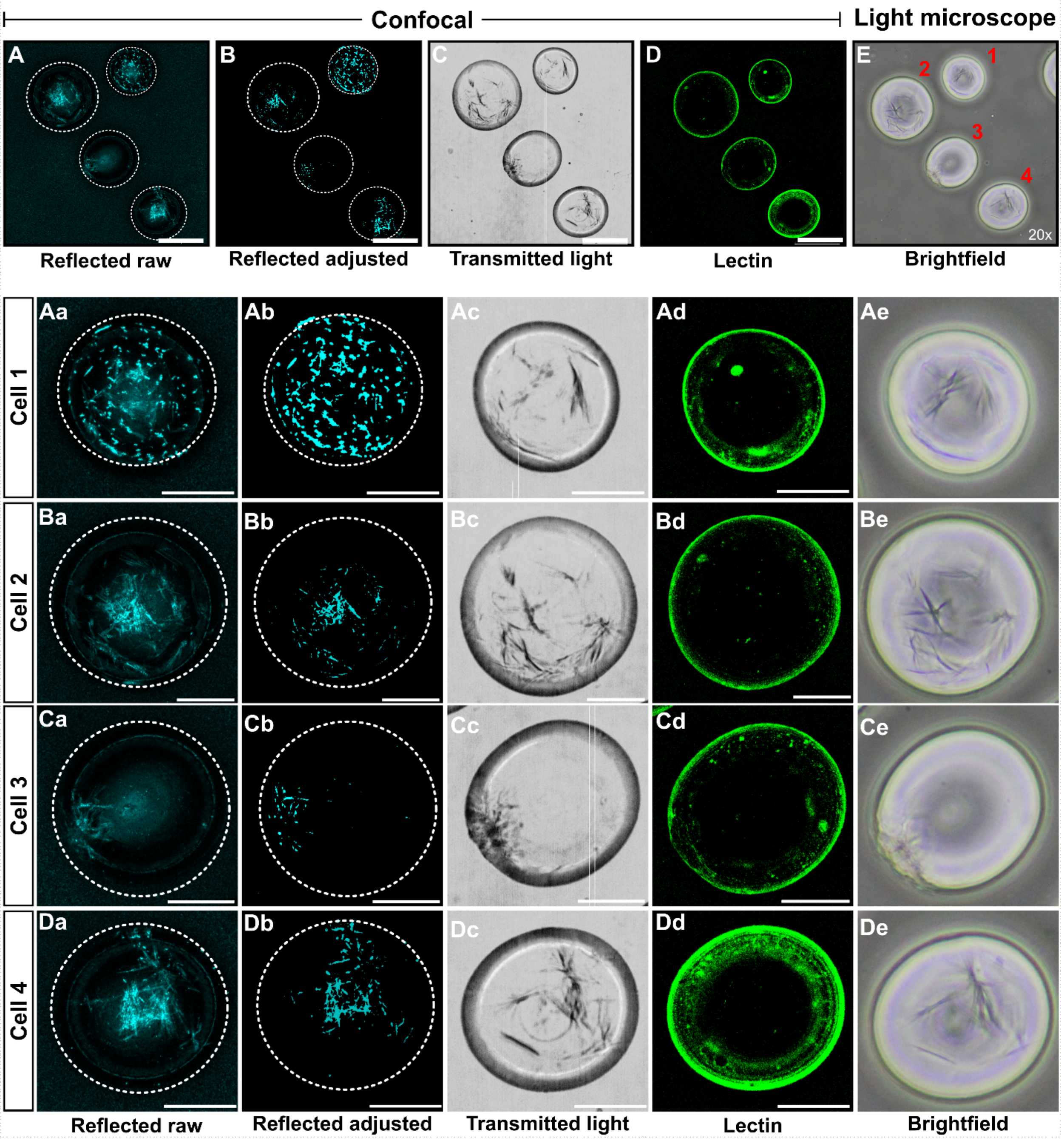

Supplementary Figure 2

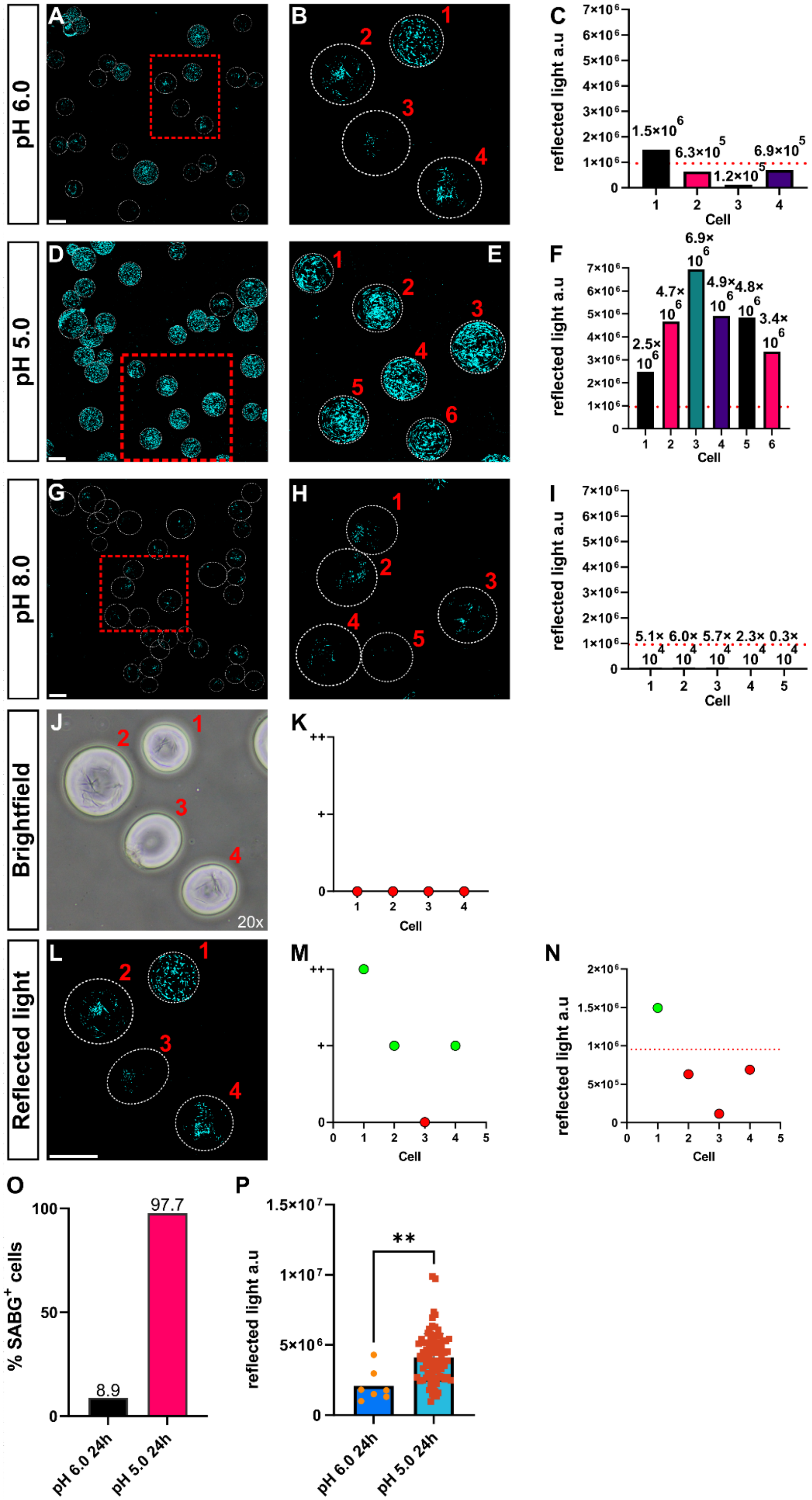

Supplementary Figure 3

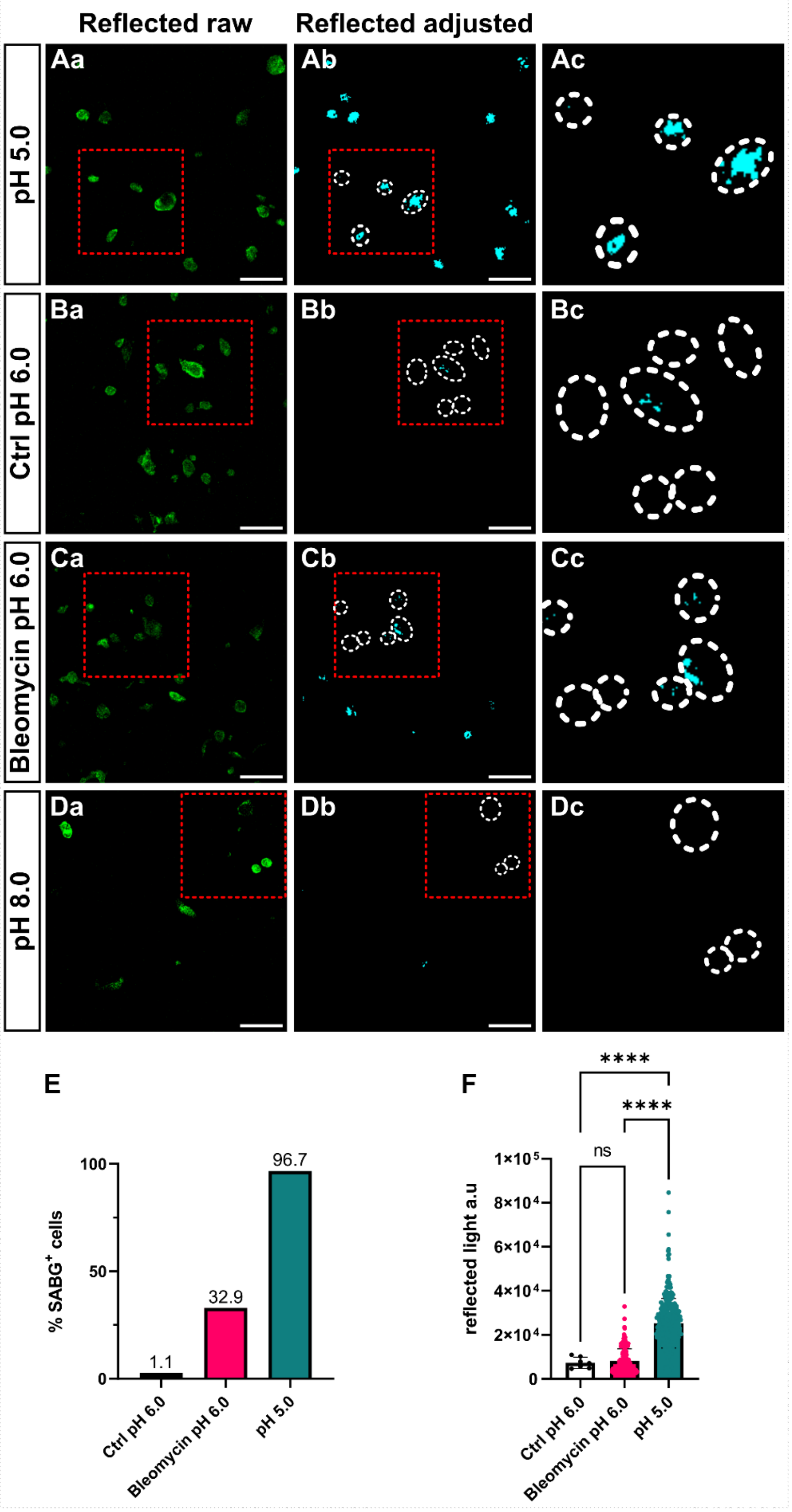

Supplementary Figure 4

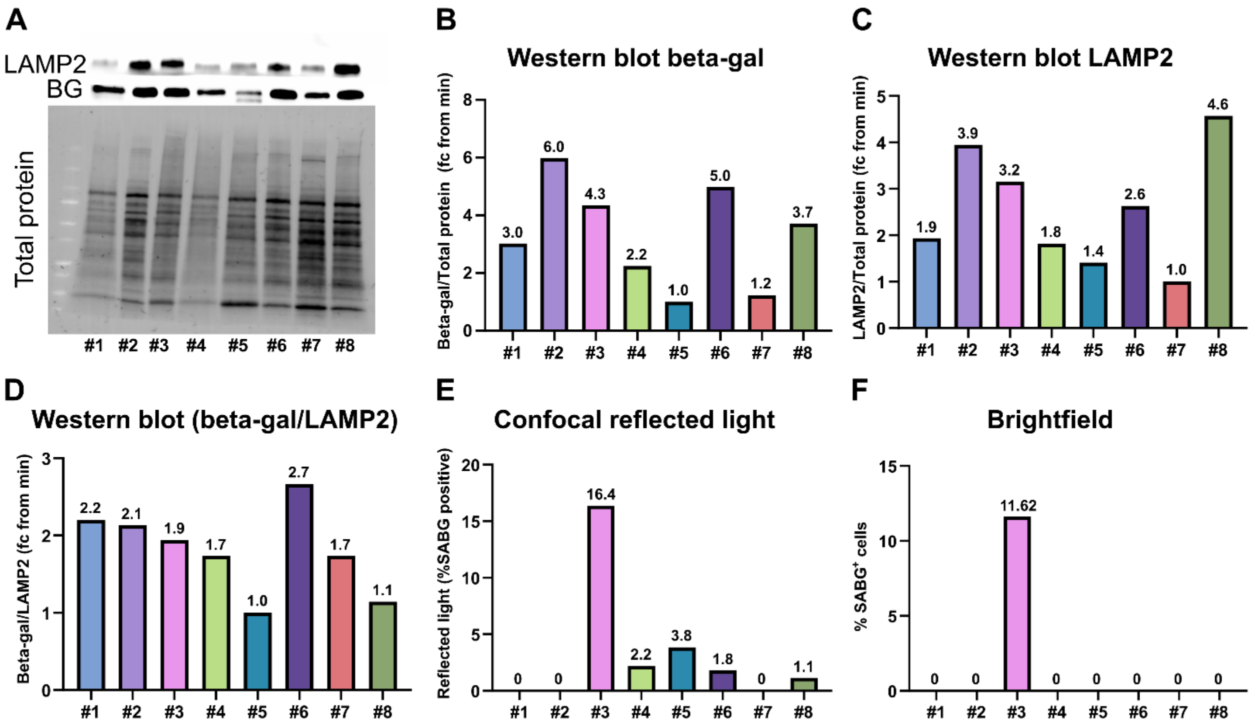

Supplementary Figure 5

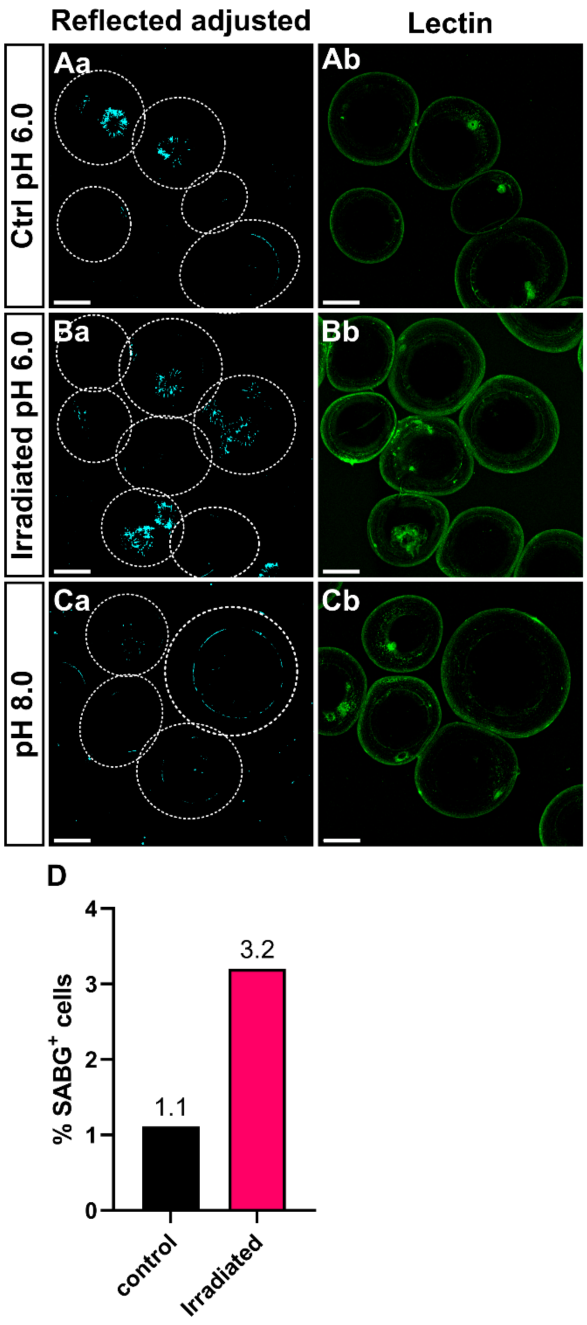

Supplementary Figure 6

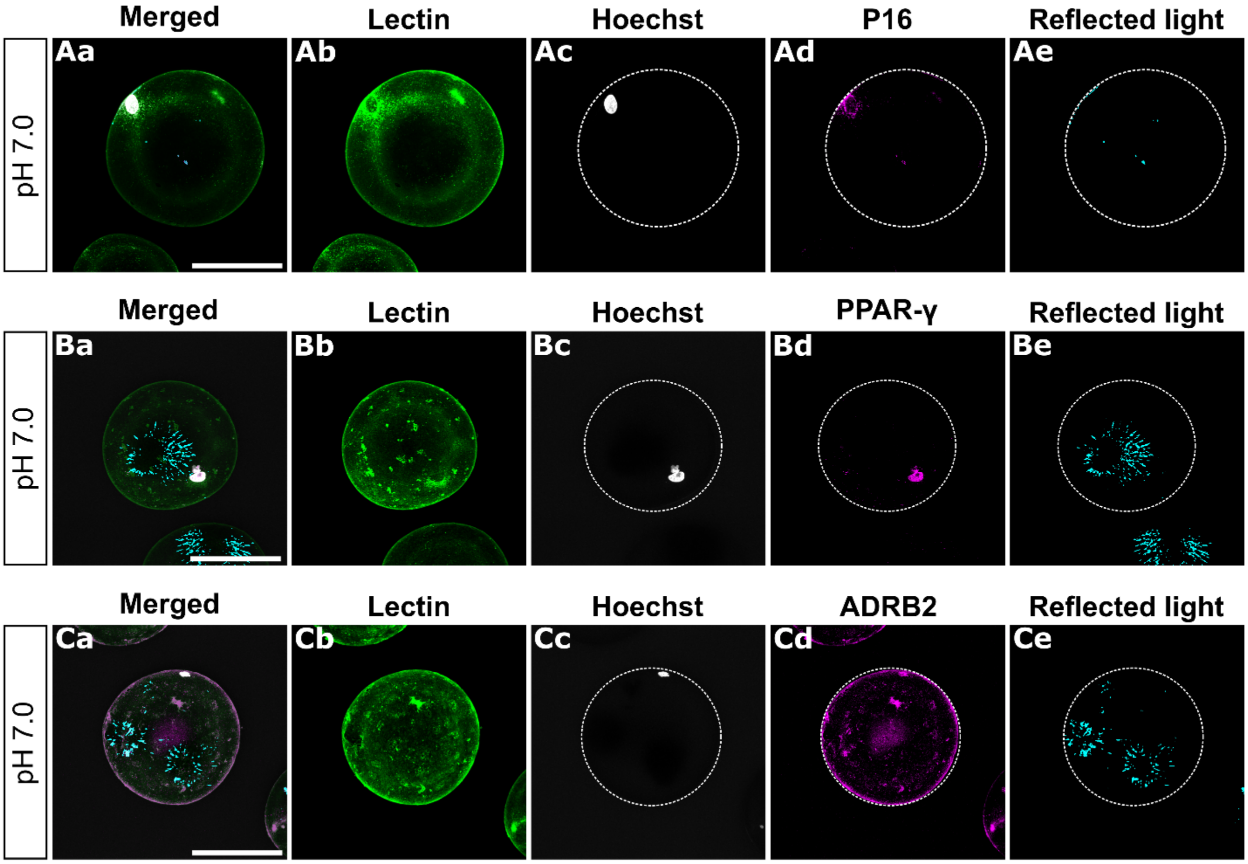

Supplement: Supplementary file 1 — Table S1. Patient cohort characteristics for experiments in Figure S1. The cohort consisted of eight people in total. Data are reported as mean and range. Figure S1. Overview of differences in X‐gal staining observed using confocal reflected light and brightfield microscopy in mouse adipocytes. Maximum image projection of four mature mouse primary adipocytes stained with X‐gal, visualized by (A) confocal raw reflected light, (B) confocal reflected light adjusted, (C) confocal transmitted light and (D) cell membrane stain lectin. (E) Brightfield image of the same four mature mouse primary adipocytes. (Aa–Ee) Digitally zoomed images of cells from (A–D) showing raw reflected light, reflected light adjusted, transmitted light, lectin and brightfield. Dotted white lines indicate cell perimeter. Red numbers indicate cell numbers. All images were captured using a 20x objective. White scalebars in A, B, C and D are equivalent to 100 μm, scalebars in Aa‐Dd are equivalent to 50 μm. Figure S2. Establishing pH controls for the thresholding of SABG positive mouse adipocytes. (A) Cells incubated with X‐gal at pH 6.0 to detect SABG activity. (B) Digitally zoomed in image of red box in (A), showing cell perimeters and (C) quantification of confocal reflected light intensity per cell. (D) Cells incubated with X‐gal at pH 5.0 to detect total beta‐galactosidase activity. (E) Digitally zoomed in image of red box in (D), with cell perimeters and (F) reflected light intensities per cell. (G) Cells incubated with X‐gal at pH 8.0 to measure background reflected light for thresholding. (H) Digitally zoomed in image of red box in (G), with cell perimeters and (I) reflected light intensities per cell. Signal at pH 8.0 stems from background reflected light and is the basis for the threshold indicated by the dotted red lines in (C), (F), (I), and (N). (J) Cells from Figure S1 viewed using brightfield microscopy. (K) Scoring of cells in (J) based on blue precipitates. (L) Reflected light maximum [file ACEL-23-e14295-s001.pdf]
